# Supplementary material for: Taphonomic and Diagenetic Pathways to Protein Preservation, Part II: The Case of Brachylophosaurus canadensis Specimen MOR 2598
Source: Biology (Basel). 2022 Aug 5;11(8):1177. doi: 10.3390/biology11081177 (PMC9404959; doi:10.3390/biology11081177)
Supplement: Supplementary file 1 [file biology-11-01177-s001.zip › Supplementary Materials narrative.pdf]

SUPPORTING INFORMATION

Biology

Taphonomic and diagenetic pathways to protein preservation, part II: the case of *Brachylophosaurus canadensis* specimen MOR 2598

Paul V. Ullmann[1,*], Richard D. Ash[2], John B. Scannella[3,4]

[1]Department of Geology, Rowan University, Glassboro, NJ 08028, USA

[2]Department of Geology, University of Maryland, College Park, MD 20742, USA

[3]Museum of the Rockies, Montana State University, Bozeman, MT 59717, USA

[4]Department of Earth Sciences, Montana State University, Bozeman, MT 59717, USA

*Correspondence: ullmann@rowan.edu

This supplement provides: (1) additional details on our LA-ICPMS methods; (2) discussion of potential tetrad effects in the left tibia of MOR 2598; (3) discussion of potential sequestration process that may have limited REE availability to the bones of MOR 2598; (4) discussion of potential causes for the peculiar shapes of Sc and U profiles in the left tibia of MOR 2598; (5) sources for environmental data in Figure 5 of the main text, and; (6) additional data on the trace element composition of the left tibia of MOR 2598 (Figures S1, S2). Raw transect data are provided separately in Data S1 as an Excel XLSX file.

LA-ICPMS Methodology

LA-ICPMS analyses were conducted using a New Wave UP-213 (213 nm wavelength) Nd:YAG laser coupled to a Finnigan Element2 ICPMS at the University of Maryland. The laser was operated at 2–3 J/cm^2 and a pulse rate of 7 Hz. Transect data were collected using a laser diameter of 30 μm moving at a scan speed of 50 μm/s, and background collection was performed prior to each reading for 20 s. NIST 610 glass was used as an external standard and elemental concentrations were calculated based on normalization to 55.8% CaO in bone apatite.

Potential Tetrad Effects in MOR 2598

Most REE uptake behavior in natural systems can be attributed to charge and radius controls on ion reactions (so-called CHARAC behavior; [1]). Non-CHARAC behavior, in which the behavior of REE ions is also influenced by the configuration of their outer electron shell [2], can produce tetrad effects, including in fossil bones (e.g., [3,4]). Spider diagrams of NASC-

normalized REE concentrations in the tibia of MOR 2598 exhibit subtle yet distinct peaks and/or deflections at europium (Eu) and holmium (Ho) (Figures 3B and 4). These diagrams are not developed into strong M-shaped profiles, but the regular deflections are still apparent. Abundant, positive Y/Ho anomalies in the external and internal cortices (Figure S1) may also be attributable to tetrad effects during uptake [4]. According to Herwartz et al. [4], preferential uptake of LREE in the external cortex may also contribute to these patterns. Abundant evidence of for fractionation in MOR 2598 (e.g., typical intra-bone fractionation trends in the by-laser-run spider diagram [Figure 4B in the main text], low concentrations of MREE and elements with low-moderate diffusivities in the middle cortex [Figure 2A,B in the main text, Data S1]) implies that fractionation was likely a major control on the development of the high Y/Ho ratios, but the clear peaks/deflections at Eu and Ho appear more likely attributable to strong tetrad effects during uptake.

Processes Which Potentially Limited REE Availability

Complexation of dissolved REE with carbonate anions is common in natural waters [5,6], especially in coastal fresh and brackish waters [7,8] due to chemical weathering [9] and the decomposition of abundant organic matter in coastal sediments [10]. Given the geologic and geochemical indications (described herein) that MOR 2598 was buried in an estuarine channel, it is therefore plausible that extensive complexation may have partially suppressed the availability of trace elements during early diagenesis. Humic acids released during the decay of plant debris in the surrounding sediments and of any residual dinosaur tissues (e.g., muscle) may also have formed complexes with dissolved REE [11], further limiting their availability for uptake by the

bone. Dissolved phosphates released from decaying organic matter [12–14] may also have scavenged REE ions from solution via coprecipitation within trace secondary phosphates in the surrounding sediments (cf. [15,16]). We were not able to test quarry sediments for trace phosphatic phases in this study, but they are almost certainly present as lowland/coastal watersheds generally contain more phosphate than upstream regions [13,17]. In the absence of such direct tests, these potential sequestration processes (carbonate complexation and/or coprecipitation with phosphate) remain speculative, but the depositional setting implies that each may have contributed to limiting the supply of trace elements within surrounding early-diagenetic pore fluids.

Potential Explanations for Sc and U Profile Shapes

Sc and U are the only trace elements examined in MOR 2598 to exhibit broad peaks in concentrations across the middle cortex (Figure 2B in the main text). The causes(s) of this are unclear. Although the internal "boundary" of their peaks (near 25 mm) spatially corresponds with a transition in histologic structure from more random Haversian bone with numerous secondary osteons (internal cortex) to denser, more organized, fibrolamellar, laminar bone (middle cortex) (see thick section inlays in Figure 2 of the main text), there is no similar apparent transition in histologic microstructure between the middle and external cortices. Lack of permineralization implies that there were no spatial contrasts in fluid flow potential either. Therefore, the only potential explanation we can advance is that simultaneous pore fluid diffusion toward the middle cortex from both outside the bone and the medullary cavity during early diagenesis may have caused heavily-fractionated groundwaters to 'pool' in the middle

cortex, which over time led to the development of elevated concentrations of U and other elements still abundant in solution (i.e., Sc) in this region of the cortex.

Sources for Environmental Data in Figure 5

Literature data for environmental samples in Figure 5A of the main text are as follows: river waters (green field; [18–37]); suspended river loads (dull pink field; [19,38]); groundwaters (bright pink field; [24,39–47]); lakes (purple field; [24,27,28,31,40,43,48,49]); estuaries (yellow field; [20,46,50–52]); coastal waters (light blue field; [18,20,21,32,53,54]); seawater (dark blue field; [55–73]); sea floor particles (gray field; [59,67]); marine pore fluids (orange field; [53,71,74,75]).

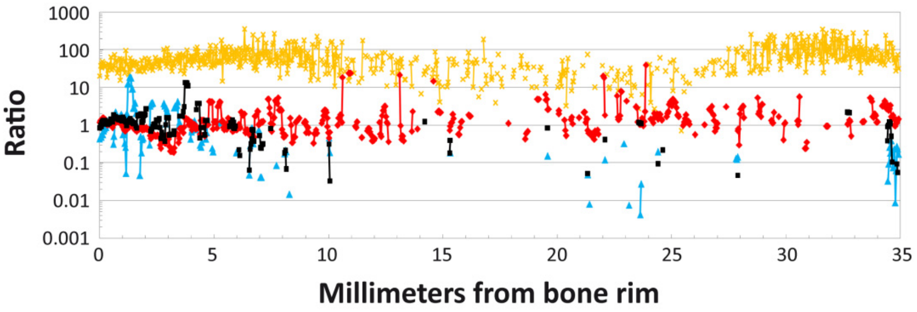

Figure S1. Intra-bone patterns of $(Ce/Ce^*)_N$, $(Ce/Ce^{**})_N$, and $(La/La^*)_N$ anomalies and Y/Ho ratios within the tibia of MOR 2598. $(Ce/Ce^*)_N$ values (red curves), $(Ce/Ce^{**})_N$ values (black curves), and $(La/La^*)_N$ anomalies (blue curves) were calculated as outlined in the Materials and Methods. Y/Ho ratio data are presented as the orange curve. Absence of $(Ce/Ce^*)_N$, $(Ce/Ce^{**})_N$, and $(La/La^*)_N$ anomalies occurs at 1.0.

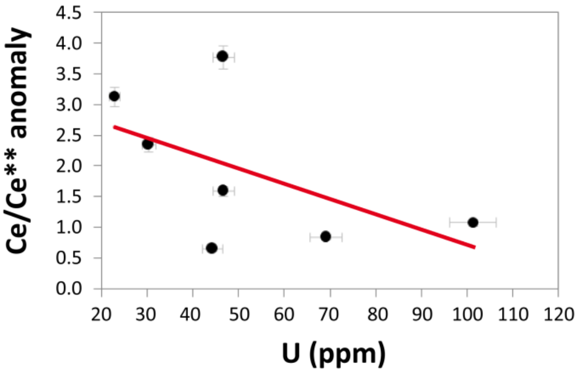

Figure S2. Cerium anomaly $(Ce/Ce^{**})_N$ values plotted against uranium (U) concentrations in the tibia of MOR 2598. Absence of the cerium anomaly is at a value of 1.0. Error bars, in gray, are based on analytical reproducibility of ± 5%. The trendline in red has a very poor fit ($r^2 = 0.29$), suggesting different timescales for uptake of U and Ce (according to [76]).

REFERENCES

1) Bau, M. Controls on the fractionation of isovalent trace elements in magmatic and aqueous systems: evidence from Y/Ho, Zr/Hf, and lanthanide tetrad effect. *Contrib. Mineral. Petr.* **1996**, *123*, 323–333.

2) Hinz, E.A.; Kohn, M.J. The effect of tissue structure and soil chemistry on trace element uptake in fossils. *Geochim. Cosmochim. Ac.* **2010**, *74*, 3213–3231.

3) Grandstaff, D.E.; Terry, D.O. Rare earth element composition of Paleogene vertebrate fossils from Toadstool Geologic Park, Nebraska, USA. *Appl. Geochem.* **2009**, *24*, 733–745.

4) Herwartz, D.; Tütken, T.; Jochum, K.P.; Sander, P.M. Rare earth element systematics of fossil bone revealed by LA-ICPMS analysis. *Geochim. Cosmochim. Ac.* **2013**, *103*, 161–183.

5) Cantrell, K.J.; Byrne, R.H. Rare earth element complexation by carbonate and oxalate ions. *Geochim. Cosmochim. Ac.* **1987**, *51*, 597–605.

6) Luo, Y.-R.; Byrne, R.H. Carbonate complexation of yttrium and the rare earth elements in natural waters. *Geochim. Cosmochim. Ac.* **2004**, *68*, 691–699.

7) Livingstone, D.A. Chemical composition of rivers and lakes. In *Data of Geochemistry*; Fleischer, M., Ed.; USGS: Washington, DC, USA, **1963**; pp. G1–G64.

8) Zhang, J.; Létolle, R.; Jusserand, C. Major element chemistry of the Huanghe (Yellow River), China – weathering processes and chemical fluxes. *J. Hydrol.* **1995**, *168*, 173–203.

9) Stallard, R.F.; Edmond, J.M. Geochemistry of the Amazon 3. Weathering chemistry and limits to dissolved inputs. *J. Geophys. Res.* **1987**, *92*, 8293–8302.

10) Berner, R.A. Calcium carbonate concretions formed by the decomposition of organic matter. *Science* **1968**, *159*, 195–197.

11) Pourret, O.; Davranche, M.; Gruau, G.; Dia, A. Rare earth elements complexation with humic acid. *Chem. Geol.* **2007**, *243*, 128–141.

12) Allison, P.A. Konservat-Lagerstätten: cause and classification. *Paleobiology* **1988**, *14*, 331–344.

13) Föllmi, K.B. The phosphorous cycle, phosphogenesis and marine phosphate-rich deposits. *Earth-Sci. Rev.* **1996**, *40*, 55–124.

14) Wang, L.; Liu, Q.; Hu, C.; Liang, R.; Qiu, J.; Wang, Y. Phosphorous release during decomposition of the submerged macrophyte *Potamogeton crispus*. *Limnology* **2018**, *19*, 355–366.

15) Byrne, R.H.; Liu, X.; Schijf, J. The influence of phosphate coprecipitation on rare earth distributions in natural waters. *Geochim. Cosmochim. Ac*. **1996**, *60*, 3341–3346.

16) Liu, X.; Byrne, R.H.; Schijf, J. Comparative coprecipitation of phosphate and arsenate with yttrium and the rare earths: the influence of solution complexation. *J. Solution Chem.* **1997**, *26*, 1187–1198.

17) Brunet, R.-C.; Astin, K.B. Variation in phosphorus flux during a hydrological season: the River Ardour. *Wat. Res.* **1998**, *32*, 547–558.

18) Hoyle, J.; Elderfield, H.; Gledhill, A.; Greaves, M. The behaviour of the rare earth elements during mixing of river and sea waters. *Geochim. Cosmochim. Ac*. **1984**, *48*, 143–149.

19) Goldstein, S.J.; Jacobsen, S.B. Rare earth elements in river waters. *Earth Planet. Sc. Lett.* **1988**, *89*, 35–47.

20) Elderfield, H.; Upstill-Goddard, R.; Sholkovitz, E.R. The rare earth elements in rivers, estuaries, and coastal seas and their significance to the composition of ocean waters. *Geochim. Cosmochim. Ac*. **1990**, *54*, 971–991.

21) Bau, M.; Dulski P. Anthropogenic origin of positive gadolinium anomalies in river waters. *Earth Planet. Sc. Lett*. **1996**, *143*, 245–255.

22) Biddau, R.; Cidu, R.; Frau, F. Rare earth elements in waters from the albitite-bearing granodiorites of Central Sardinia, Italy. *Chem. Geol*. **2002**, *182*, 1–14.

23) Åström, M.; Corin, N. Distribution of rare earth elements in anionic, cationic and particulate fractions in boreal humus-rich streams affected by acid sulphate soils. *Water Res.* **2003**, *37*, 273–280.

24) Bwire Ojiambo, S.; Lyons, W.B.; Welch, K.A.; Poreda, R.J.; Johannesson, K.H. Strontium isotopes and rare earth elements as tracers of groundwater-lake water interactions, Lake Naivasha, Kenya. *Appl. Geochem.* **2003**, *18*, 1789–1805.

25) Tang, J.; Johannesson, K.H. Speciation of rare earth elements in natural terrestrial waters: assessing the role of dissolved organic matter from the modeling approach. *Geochim. Cosmochim. Ac*. **2003**, *67*, 2321–2339.

26) Centeno, L.M.; Faure, G.; Lee, G.; Talnagi, J. Fractionation of chemical elements including the REEs and ^{226}Ra in stream contaminated with coal-mine effluent. *Appl. Geochem.* **2004**, *19*, 1085–1095.

27) Johannesson, K.H.; Tang, J.; Daniels, J.M.; Bounds, W.J.; Burdige, D.J. Rare earth element concentrations and speciation in organic-rich blackwaters of the Great Dismal Swamp, Virginia, USA. *Chem. Geol*. **2004**, *209*, 271–294.

28) Gammons, C.H.; Wood, S.A.; Pedrozo, F.; Varekamp, J.C.; Nelson, B.J.; Shope, C.L.; Baffico, G. Hydrogeochemistry and rare earth element behavior in a volcanically acidified watershed in Patagonia, Argentina. *Chem. Geol.* **2005**, *222*, 249–267.

29) Gammons, C.H.; Wood, S.A.; Nimick, D.A. Diel behavior of rare earth elements in a mountain stream with acidic to neutral pH. *Geochim. Cosmochim. Ac.* **2005**, *69*, 3747–3758.

30) Barroux, G.; Sonke, J.E.; Boaventura, G.; Viers, J.; Godderis, Y.; Bonnet, M.-P.; Sondag, F.; Gardoll, S.; Lagane, C.; Seyler, P. Seasonal dissolved rare earth element dynamics of the Amazon River main stem, its tributaries, and the Curuaí floodplain. *Geochem. Geophy. Geosy.* **2006**, *7*, Q12005.

31) Bau, M.; Knappe, A.; Dulski, P. Anthropogenic gadolinium as a micropollutant in river waters in Pennsylvania and in Lake Erie, northeastern United States. *Chem. Erde-Geochem.* **2006**, *66*, 143–152.

32) Kulaksiz, S.; Bau, M. Contrasting behaviour of anthropogenic gadolinium and natural rare earth elements in estuaries and the gadolinium input into the North Sea. *Earth Planet. Sc. Lett.* **2007**, *260*, 361–371.

33) Kulaksiz, S.; Bau, M. Anthropogenic gadolinium as a microcontaminant in tap water used as drinking water in urban areas and megacities. *Appl. Geochem.* **2011**, *26*, 1877–1885.

34) Pokrovsky, O.S.; Viers, J.; Shirokova, L.S.; Shevchenko, V.P.; Filipov, A.S.; Dupré, B. Dissolved, suspended, and colloidal fluxes of organic carbon, major and trace elements in the Severnaya Dvina River and its tributary. *Chem. Geol.* **2010**, *273*, 136–149.

35) Censi, P.; Sposito, F.; Inguaggiato, C.; Zuddas, P.; Inguaggiato, S.; Venturi, M. Zr, Hf and REE distribution in river water under different ionic strength conditions. *Sci. Total Environ.* **2018**, *645*, 837–853.

36) Kalender, L.; Aytimur, G. REE geochemistry of Euphrates River, Turkey. *J. Chem.* **2016**, *1*, 1–13.

37) Smith, C.; Liu, X.-M. Spatial and temporal distribution of rare earth elements in the Neuse River, North Carolina. *Chem. Geol.* **2018**, *488*, 34–43.

38) Merschel, G.; Bau, M.; Schmidt, K.; Münker, C.; Dantas, E.L. Hafnium and neodymium isotopes and REY distribution in the truly dissolved, nanoparticulate/colloidal and suspended loads of rivers in the Amazon Basin, Brazil. *Geochim. Cosmochim. Ac.* **2017**, *213*, 383–399.

39) Smedley, P.L. The geochemistry of rare earth elements in groundwater from the Carnmenellis area, southwest England. *Geochim. Cosmochim. Ac.* **1991**, *55*, 2767–2779.

40) Johannesson, K.H.; Lyons, W.B.; Stetzenbach, K.J.; Byrne, R.H. The solubility control of rare earth elements in natural terrestrial waters and the significance of PO_4^{3-} and CO_3^{2-} in

limiting dissolved rare earth concentrations: a review of recent information. *Aquat. Geochem.* **1995**, *1*, 157–173.

41) Johannesson, K.H.; Stetzenbach, K.J.; Hodge, V.F. Rare earth elements as geochemical tracers of regional groundwater mixing. *Geochim. Cosmochim. Ac.* **1997**, *61*, 3605–3618.

42) Johannesson, K.H.; Farnham, I.M.; Guo, C.; Stetzenbach, K.J. Rare earth element fractionation and concentration variations along a groundwater flow path within a shallow, basin-fill aquifer, southern Nevada, USA. *Geochim. Cosmochim. Ac.* **1999**, *63*, 2697–2708.

43) Leybourne, M.I.; Goodfellow, W.D.; Boyle, D.R.; Hall, G.M. Rapid development of negative Ce anomalies in surface waters and contrasting REE patterns in groundwaters associated with Zn-Pb massive sulphide deposits. *Appl. Geochem.* **2000**, *15*, 695–723.

44) Tang, J.; Johannesson, K.H. Controls on the geochemistry of rare earth elements along a groundwater flow path in the Carrizo Sand aquifer, Texas, USA. *Chem. Geol.* **2006**, *225*, 156–171.

45) Esmaeili-Vardanjani, M.; Shamsipour-Dehkordi, R.; Eslami, A.; Moosaei, F.; Pazand, K. A study of differentiation pattern and rare earth elements migration in geochemical and hydrogeochemical environments of Airekan and Cheshmeh Shotori areas (Central Iran). *Environ. Earth Sci.* **2013**, *68*, 719-732.

46) Chevis, D.A.; Johannesson, K.H.; Burdige, D.J.; Tang, J.; Moran, S.B.; Kelly, R.P. Submarine groundwater discharge of rare earth elements to a tidally-mixed estuary in Southern Rhode Island. *Chem. Geol.* **2015**, *397*, 128–142.

47) Liu, H.; Guo, H.; Xing, L.; Zhan, Y.; Li, F.; Shao, J.; Niu, H.; Liang, X.; Li, C. Geochemical behaviors of rare earth elements in groundwater along a flow path in the North China Plain. *J. Asian Earth Sci.* **2016**, *117*, 33–51.

48) Johannesson, K.H.; Lyons, W.B. Rare-earth element geochemistry of Colour Lake, an acidic freshwater lake on Axel Heiberg Island, Northwest Territories, Canada. *Chem. Geol.* **1995**, *119*, 209–223.

49) De Carlo, E.H.; Green W.J. Rare earth elements in the water column of Lake Vanda, McMurdo Dry Valleys, Antarctica. *Geochim. Cosmochim. Ac.* **2002**, *66*, 1323–1333.

50) Sholkovitz, E.R. The geochemistry of rare earth elements in the Amazon River estuary. *Geochim. Cosmochim. Ac.* **1993**, *57*, 2181–2190.

51) Nozaki, Y.; Lerche, D.; Alibo, D.S.; Tsutsumi, M. Dissolved indium and rare earth elements in three Japanese rivers and Tokyo Bay: evidence for anthropogenic Gd and In. *Geochim. Cosmochim. Ac.* **2000**, *64*, 3975–3982.

52) Rousseau, T.C.C.; Sonke, J.E.; Chmeleff, J.; van Beek, P.; Souhat, M.; Boaventura, G.; Seyler, P.; Jeandel, C. Rapid neodymium release to marine waters from lithogenic sediments in the Amazon estuary. *Nat. Commun.* **2015**, *6*, 7592.

53) Elderfield, H.; Sholkovitz, E.R. Rare earth elements in the pore waters of reducing nearshore sediments. *Earth Planet. Sc. Lett*. **1987**, *82*, 280–288.

54) Bayon, G.; Birot, D.; Ruffine, L.; Caprais, J.-C.; Ponzevera, E.; Bollinger, C.; Donval, J.-P.; Charlou, J.-L.; Voisset, M.; Grimaud, S. Evidence for intense REE scavenging at cold seeps from the Niger Delta margin. *Earth Planet. Sc. Lett*. **2011**, *312*, 443–452.

55) Elderfield, H.; Greaves, M.J. The rare earth elements in seawater. *Nature* **1982**, *296*, 214–219.

56) de Baar, H.J.W.; Bacon, M.P.; Brewer, P.G. Rare-earth distributions with a positive Ce anomaly in the Western North Atlantic Ocean. *Nature* **1983**, *301*, 324–327.

57) German, C.R.; Holliday, B.P.; Elderfield, H. Redox cycling of rare earth elements in the suboxic zone of the Black Sea. *Geochim. Cosmochim. Ac*. **1991**, *55*, 3553–3558.

58) Piepgras, D.J.; Jacobsen, S.B. The behavior of rare earth elements in seawater: precise determination of variations in the North Pacific water column. *Geochim. Cosmochim. Ac.* **1992**, *56*, 1851–1862.

59) Sholkovitz, E.R.; Landing, W.M.; Lewis, B.L. Ocean particle chemistry: the fractionation of rare earth elements between suspended particles and seawater. *Geochim. Cosmochim. Ac.* **1994**, *58*, 1567–1579.

60) German, C.R.; Masuzawa, T.; Greaves, M.J.; Elderfield, H.; Edmond, J.M. Dissolved rare earth elements in the Southern Ocean: cerium oxidation and the influence of hydrography. *Geochim. Cosmochim. Ac*. **1995**, *59*, 1551–1558.

61) Zhang, J.; Nozaki, Y. Rare earth elements and yttrium in seawater: ICP-MS determinations in the East Caroline, Coral Sea, and South Fiji basins of the western South Pacific Ocean. *Geochim. Cosmochim. Ac*. **1996**, *60*, 4631–4644.

62) Hongo, Y.; Obata, H.; Alibo, D.S.; Nozaki, Y. Spatial variations of rare earth elements in North Pacific surface water. *J. Oceanogr.* **2006**, *62*, 441–455.

63) Wang, Z.-L.; Yamada, M. Geochemistry of dissolved rare earth elements in the Equatorial Pacific Ocean. *Environ. Geol.* **2007**, *52*, 779–787.

64) van de Flierdt, T.; Pahnke, K.; Amakawa, H.; Andersson, P.; Basak, C.; Coles, B.; Colin, C.; Crocket, K.; Frank, M.; Frank, N.; Goldstein, S.L.; Goswami, V.; Haley, B.A.; Hathorne, E.C.; Hemming, S.R.; Henderson, G.M.; Jeandel, C.; Jones, K.; Kreissig, K.; Lacan, F.; Lambelet, M.; Martin, E.E.; Newkirk, D.R.; Obata, H.; Pena, L.; Piotrowski, A.M.; Pradoux,

C.; Scher, H.D.; Schöberg, H.; Singh, S.K.; Stichel, T.; Tazoe, H.; Vance, D.; Yang, J. GEOTRACES intercalibration of neodymium isotopes and rare earth element concentrations in seawater and suspended particles. Part 1: reproducibility of results for the international intercomparison. *Limnol. Oceanogr. Methods* **2012**, *10*, 234–251.

65) Grenier, M.; Jeandel, C.; Lacan, F.; Vance, D.; Venchiarutti, C.; Cros, A.; Cravatte, S. From the subtropics to the central equatorial Pacific Ocean: neodymium isotopic composition and rare earth element concentration variations. *J. Geophys. Res.-Oceans* **2013**, *118*, 592–618.

66) Jeandel, C.; Delattre, H.; Grenier, M.; Pradoux, C.; Lacan, F. Rare earth element concentrations and Nd isotopes in the Southeast Pacific Ocean. *Geochem. Geophy. Geosy.* **2013**, *14*, 328–341.

67) Garcia-Solsona, E.; Jeandel, C.; Labatut, M.; Lacan, F.; Vance, D.; Chavagnac, V.; Pradoux, C. Rare earth elements and Nd isotopes tracing water mass mixing and particle-seawater interactions in the SE Atlantic. *Geochim. Cosmochim. Ac*. **2014**, *125*, 351–372.

68) Abbott, A.N.; Haley, B.A.; McManus, J.; Reimers, C.E. The sedimentary flux of dissolved rare earth elements to the ocean. *Geochim. Cosmochim. Ac*. **2015**, *154*, 186–200.

69) Hathorne, E.C.; Stichel, T.; Brück, B.; Frank, M. Rare earth element distribution in the Atlantic sector of the Southern Ocean: the balance between particle scavenging and vertical supply. *Mar. Chem.* **2015**, *177*, 157–171.

70) Zheng, X.-Y.; Plancherel, Y.; Saito, M.A.; Scott, P.M.; Henderson, G.M. Rare earth elements (REEs) in the tropical South Atlantic and quantitative deconvolution of their non-conservative behavior. *Geochim. Cosmochim. Ac*. **2016**, *177*, 217–237.

71) Johannesson, K.H.; Palmore, C.D.; Fackrell, J.; Prouty, N.G.; Swarzenski, P.W.; Chevis, D.A.; Telfeyan, K.; White, C.D.; Burdige, D.J. Rare earth element behavior during groundwater-seawater mixing along the Kona Coast of Hawaii. *Geochim. Cosmochim. Ac*. **2017**, *198*, 229–258.

72) Osborne, A.H.; Hathorne, E.C.; Schijf, J.; Plancherel, Y.; Böning, P.; Frank, M. The potential of sedimentary foraminiferal rare earth element patterns to trace water masses in the past. *Geochem. Geophy. Geosy.* **2017**, *18*, 1550–1568.

73) de Baar, H.J.W.; Bruland, K.W.; Schijf, J.; van Heuven, S.M.A.C.; Behrens, M.K. Low cerium among the dissolved rare earth elements in the central North Pacific Ocean. *Geochim. Cosmochim. Ac*. **2018**, *236*, 5–40.

74) Haley, B.A.; Klinkhammer, G.P.; McManus, J. Rare earth elements in pore waters of marine sediments. *Geochim. Cosmochim. Ac*. **2004**, *68*, 1265–1279.

75) Kim, J.-H.; Torres, M.E.; Haley, B.A.; Kastner, M.; Pohlman, J.W.; Riedel, M.; Lee, Y.J. The effect of diagenesis and fluid migration on rare earth element distribution in fluids of the northern Cascadia accretionary margin. *Chem. Geol.* **2011**, *291*, 152–165.

76) Metzger, C.A.; Terry, D.O.; Grandstaff, D.E. Effect of paleosol formation on rare earth element signatures in fossil bone. *Geology* **2004**, *32*, 497–500.
